# Training Performance Assessment for Intracranial Aneurysm Clipping Surgery Using a Patient-Specific Mixed-Reality Simulator: A Learning Curve Study

**DOI:** 10.1227/ons.0000000000001041

**Published:** 2024-01-22

**Authors:** Miguel Cuba, Hanne Vanluchene, Michael Murek, Johannes Goldberg, Mandy D. Müller, Matteo Montalbetti, Katharina Janosovits, Thomas Rhomberg, David Zhang, Andreas Raabe, Fredrick J. Joseph, David Bervini

**Affiliations:** ‡ Image Guided Therapy, ARTORG Center for Biomedical Engineering Research, University of Bern, Bern, Switzerland;; § Department of Neurosurgery, Inselspital Bern University Hospital, University of Bern, Bern, Switzerland

**Keywords:** Aneurysm clipping, Skill development, Surgical microscope, Surgical simulation, Surgical training

## Abstract

**BACKGROUND AND OBJECTIVES:**

The value of simulation-based training in medicine and surgery has been widely demonstrated. This study investigates the introduction and use of a new mixed-reality neurosurgical simulator in aneurysm clipping surgery, focusing on the learning curve and performance improvement.

**METHODS:**

Five true-scale craniotomy head models replicating patient-specific neuroanatomy, along with a mixed-reality simulator, a neurosurgical microscope, and a set of microsurgical instruments and clips, were used in the operation theater to simulate aneurysm microsurgery. Six neurosurgical residents participated in five video-recorded simulation sessions over 4 months. Complementary learning modalities were implemented between sessions. Thereafter, three blinded analysts reported on residents' use of the microscope, quality of manipulation, aneurysm occlusion, clipping techniques, and aneurysm rupture. Data were also captured regarding training time and clipping attempts.

**RESULTS:**

Over the course of training, clipping time and number of clipping attempts decreased significantly (*P* = .018, *P* = .032) and the microscopic skills improved (*P* = .027). Quality of manipulation and aneurysm occlusion scoring improved initially although the trend was interrupted because the spacing between sessions increased. Significant differences in clipping time and attempts were observed between the most and least challenging patient models (*P* = .005, *P* = .0125). The least challenging models presented higher rates of occlusion based on indocyanine green angiography evaluation from the simulator.

**CONCLUSION:**

The intracranial aneurysm clipping learning curve can be improved by implementing a new mixed-reality simulator in dedicated training programs. The simulator and the models enable comprehensive training under the guidance of a mentor.

**ABBREVIATIONS:**
ICG: indocyanine green
OT: operation theater
VR: virtual reality.

The delicate and high-risk nature of intracranial aneurysm (IA) clipping surgery poses considerable challenges for residents and supervisors during practical training. This demanding environment, tightly packed schedules, long duty hours, and the limited availability of qualified practitioners^1,2^ may make it difficult to acquire the experience required for a neurosurgeon to specialize in IA surgery. As a consequence, other neurosurgery subspecialties often take precedence, and medical residents are ultimately prompted to explore alternative specialties within the realms of neurosurgery and neuroradiology, further hindering the acquisition of expertise in the field of microvascular neurosurgery.^2-4^ Furthermore, even for the trainees who decide to undertake these challenges, traditional training methods often entail a direct transition from theoretical knowledge and assisting procedures to practice on an actual patient in the operation theater (OT), where a practitioner's lack of experience may raise a patient's risk of poor treatment outcomes^5,6^

To mitigate these challenges, the consensus in the literature is that further advancements in IA treatment training are crucial and that simulation training as a supplement to conventional training could improve patient outcomes by allowing trainees to learn at their convenience on a long-term basis, yet under supervision.^7-9^ If simulation training is available, it could be beneficial especially in low-resource settings.^10^ Moreover, practitioners can use simulation tools to experiment with novel treatment approaches for complex IA cases in specific patients.

IA training must involve responses that include haptic feedback and rapidly changing aneurysm's morphological properties. In addition, adequate use of the surgical microscope (including fluency in focus, magnification, and perception of the depth of microanatomic structures) is a core requirement in micro- and neurosurgeries.^11-14^ Furthermore, recent advancements in microsurgical visualization technology and the need for more minimally invasive procedures and instruments have been associated with steep learning curves requiring specific training.^15-18^

Recent developments in surgical education promise to exponentially increase the access to hands-on procedure-specific training for young surgeons and maximize patient outcomes. The literature claims that virtual reality (VR) and physical simulators based on three-dimensional-printed models facilitate training under realistic conditions based on the actual surgical workflow and lead the way toward reducing the steepness of the learning curve in neurosurgery.^7,19-21^ Nonetheless, VR simulation that can fully reproduce the interaction of the surgeon with the patient and the OT facilities, such as the surgical microscope, and accurately convey realistic haptic feedback in real time remains a challenge. By contrast, a physical simulation fully compatible with microscope use can most accurately recreate the surgical environment offline, allowing residents to practice every stage of the IA clipping under realistic conditions.^7^ However, the availability of such simulators on the market is limited. While many previous studies have focused on face, content, and predictive validity,^21-26^ more research is needed to investigate their impact on the learning curve for IA treatment.

Thus, this study aims to evaluate a novel realistic software-assisted IA physical clipping simulation device (SurgTrain™, SurgeonsLab^®^ AG), with proven face, construct, predictive validity, and educational impact,^21^ as a standardized platform for training and evaluation of IA clipping skills in neurosurgery.

## METHODS

### Patient Models

**FIGURE 1.**
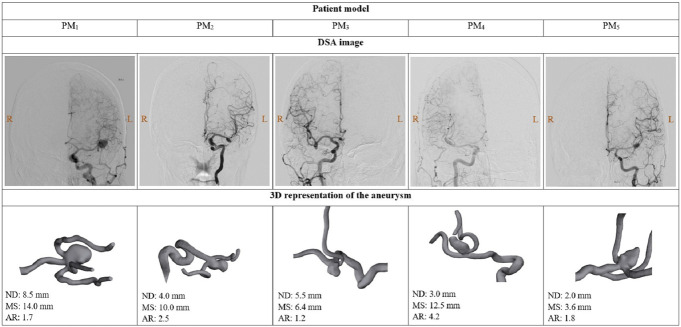
Anatomic information of the five aneurysms selected to fabricate the training models shown by DSA images and 3D representations. 3D, three-dimensional; AR, aspect ratio; DSA, digital subtraction angiography; MS, maximal size; ND, neck diameter; PM, patient model.

The study was conducted on five true-scale craniotomy head models replicating the anatomy of patients with previously treated IA. A group of left and right middle cerebral artery (MCA) bifurcation unruptured aneurysms with different morphologies were selected for the study. A detailed description of the anatomic characteristics of the selected aneurysms is presented in Figure 1. The complete models consisted of radiology-based patient-specific reconstructions of the sphenoid bone, the temporal and frontal lobe areas around the Sylvian fissure, and a set of connected high-fidelity artery models replicating the patient's neurovascular anatomy from the distal section of the internal carotid artery to the M1 and M2 segments of the MCA, including the aneurysm. The arterial replica structures were hollow, thin-walled, and dimensionally similar and had patent arterial structures that could intraoperatively rupture. As shown in **Supplemental Digital Content 1**, http://links.lww.com/ONS/B58, the IA had differential wall thicknesses from 0.10 to 1.50 mm in the aneurysm sac and from 0.30 to 0.75 mm in the neck, replicating closer to the actual IA's morphology. The hardness of the IA model was as soft as 0–10 ShA (Shore Hardness A scale), and the arterial branches were 10–20 ShA. However, the accurate IA metrological data of the patients could not be derived because of the lack of MRI data; therefore, the models were based on previous aneurysm studies.^27^ All these human-replica components were embedded into a general three-dimensional-printed craniotomized skull model. The location and dimensions of the craniotomy openings were defined under the standard pterional surgical approach as the general indication for MCA aneurysm clipping.^28^

### Simulator, Clips, and Instruments

**FIGURE 2.**
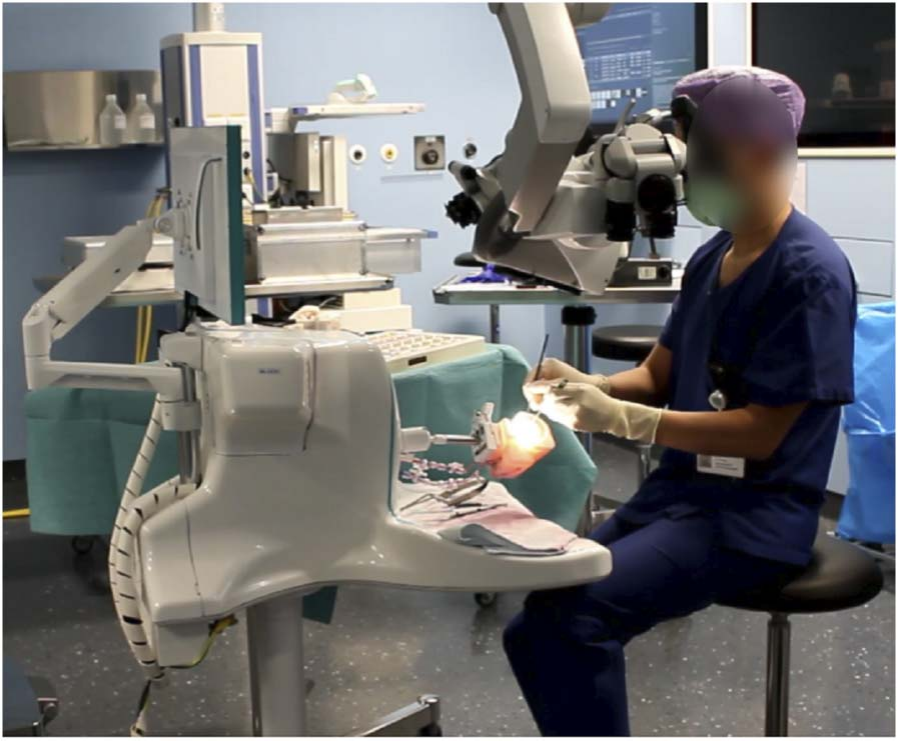
Complete operation theater setup used during simulation learning. The image shows the surgical microscope, the clip set, the simulator, and the true-scale training model held by a freely movable multiple-degree movement joint that allows for custom patient positioning. The height of the simulator can be adjusted within a range of 100–145 cm from the ground level, allowing the participants to either sit or stand as in real surgery.

The commercially available SurgTrain™ simulator (SurgeonsLab^®^ AG)^21^ was used as an interface to perfuse the models and reproduce the conditions of a true aneurysm clipping surgery. A total of 25 different titanium clips along with a clip applier (Peter Lazic GmbH) and a set of additional standard aneurysm clipping instruments, including a surgical dissector, the tip of a suction device, and a pair of forceps (Aesculap AG), were made available to the participants. The technical specifications of the surgical clips used are presented in **Supplemental Digital Content 2**, http://links.lww.com/ONS/B59. A ZEISS OPMI Pentero 800 surgical microscope (Carl Zeiss) was used with ZEISS Infrared 800 intraoperative fluorescence imaging technology. No foot control panel was used for microscope manipulation, only the mouth switch and the hand grips. The complete OT setup is illustrated in Figure 2.

### Study Participants

Six neurosurgical residents-in-training with various levels of previous experience and diverse career foci volunteered to participate in the study. Detailed information about the study participants is presented in Table 1.

**TABLE 1.** Description of the Participant's Previous Experience and Background Information

Participant	Years of neurosurgical training	No. of aneurysms clipping surgeries performed as an assistant surgeon
R_1_	6	50+
R_2_	2	1–10
R_3_	2	1–10
R_4_	3	10–20
R_5_	4	10–20
R_6_	2	1–10

R, resident.

### Study Description

**FIGURE 3.**
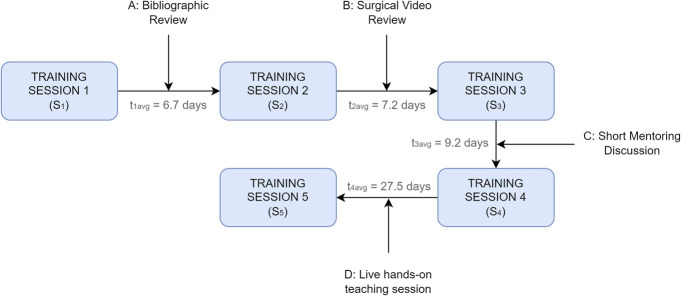
Representation of the simulation session workflow, including the training sessions, complementary learning modalities, and average time interval between sessions (t_navg_).

The complete simulation training was conducted over 4 months. First, a live introductory session was held for all the participants to familiarize themselves with using the simulator. Then, five training sessions were performed with the simulator, preceded by different complementary learning activities, as seen in Figure 3. An overview of the complementary learning methods is added in **Supplemental Digital Content 3**, http://links.lww.com/ONS/B60. An interval of 7–8 days was planned between the training sessions. However, the gap between sessions varied in a few cases because of differing work schedules. Figure 3 also shows the average interval between the sessions (t_navg_), whereas **Supplemental Digital Content 4**, http://links.lww.com/ONS/B61, summarizes each resident's time gaps in more detail.

**FIGURE 4.**
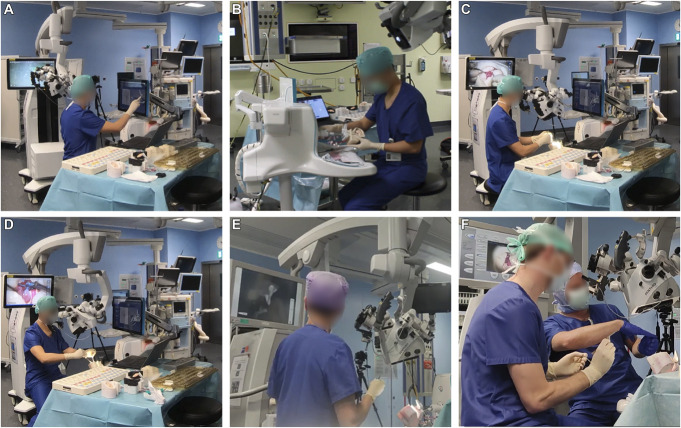
Different activities performed during the simulated training session. **A**, Radiological examination: Use of the simulator software (SurgView^TM^) to examine the patient's radiological background and the aneurysm's morphology. **B**, Patient model positioning: Model fixation to facilitate correct body posture and optimal access to the lesion. **C**, Surgical field inspection: Preliminary examination of the aneurysm's geometry and planning. **D**, Clipping: Occlusion of the aneurysm's neck (maximum 30 min, unlimited attempts). Temporary occlusion for proximal control was also allowed. **E**, Intraoperative fluorescence angiography: Evaluation of the aneurysm's occlusion, vessels' patency, and integrity using indocyanine green dye. **F**, Live hands-on teaching session from a senior neurosurgeon conducted only between sessions S_4_ and S_5_.

In each training session, each resident performed simulated aneurysm clipping on five unique patient models (PMs) in a randomized order across sessions. The residents were asked to clip each model as efficiently and quickly as possible until a suitable configuration within a maximum of 30 minutes is achieved. The simulation consisted of activities resembling real-life aneurysm surgery, illustrated in Figure 4A–4E. Figure 4F corresponds to the live hands-on teaching session.

### Blinded Expert Review

Red-green-blue (RGB) and indocyanine green (ICG) microscope videos were recorded during all training sessions. Three analysts under the supervision of expert senior cerebrovascular neurosurgeons reviewed them to evaluate the training performance based on a scoring scale shown in **Supplemental Digital Content 5**, http://links.lww.com/ONS/B62. The first and the second analysts had 2 years of experience in biomedical image and video processing and fundamental training in neurovascular surgical anatomy. The third analyst was a biomedical engineer with less than 1 year of experience in neurovascular anatomy. All analysts had been previously involved in the project on aneurysm clipping and were working on automating the microscopic video data collection, specifically focusing on the quality of clipping, aneurysm occlusion, vessel patency, and performance assessment. All residents together spent more than 135 hours training on the simulator, including the mentoring sessions, which were all recorded with the microscope. The recordings were trimmed to exclude activities without interaction and were speeded up by a factor of two. One video existed out of all training sessions (randomized) for one resident (anonymized) and for one PM (not anonymized), such a video that lasted, on average, 54 minutes. Finally, 27 hours of video was available for review, but the reviewers could speed up the video as they preferred. The videos were watched independently. The following parameters were retrieved: aneurysm occlusion (complete/incomplete); aneurysm rupture (yes/no); clipping technique (simple/multiple intersecting/multiple parallel); quality of manipulation (3-point Likert scale): combination of surehandedness, respect for tissue, and ability to expose the aneurysm during manipulation; and use of the microscope (3-point Likert scale): ability to optimally adjust the position, zoom, focus, and optical parameters of the microscope and adapt them to the circumstances of surgery.

### Data Collection

In addition, training data were collected during the training sessions in a dedicated database (REDCap 12.5.2, Vanderbilt University) during the simulation: clipping time and clipping attempts: the number of clipping attempts, where attempts are defined as modifications in the clipping technique, selection of new clips, or significant repositioning of the clips during the procedure.

### Statistical Analysis

All data were processed using Python. The scores were visualized through box and bar plots, grouping PMs and participants per training session to get an overview of the progression over time. Likert-scale scores were averaged over analysts before statistical visualization. Aneurysm occlusion, rupture, and use of clipping techniques were determined by majority voting among analysts. To check progression over time, linear regression is fitted on the mean of the performance indicators. Comparison between groups, when considered, is performed by two-sample *t*-tests. A level of significance of .05 is used.

### Ethical Disclosure

The state's Ethics Committee approved the method of this study and the use of the patient data sets. The participants and any identifiable individuals consented to publication of his/her image.

## RESULTS

### Performance vs Time

**FIGURE 5.**
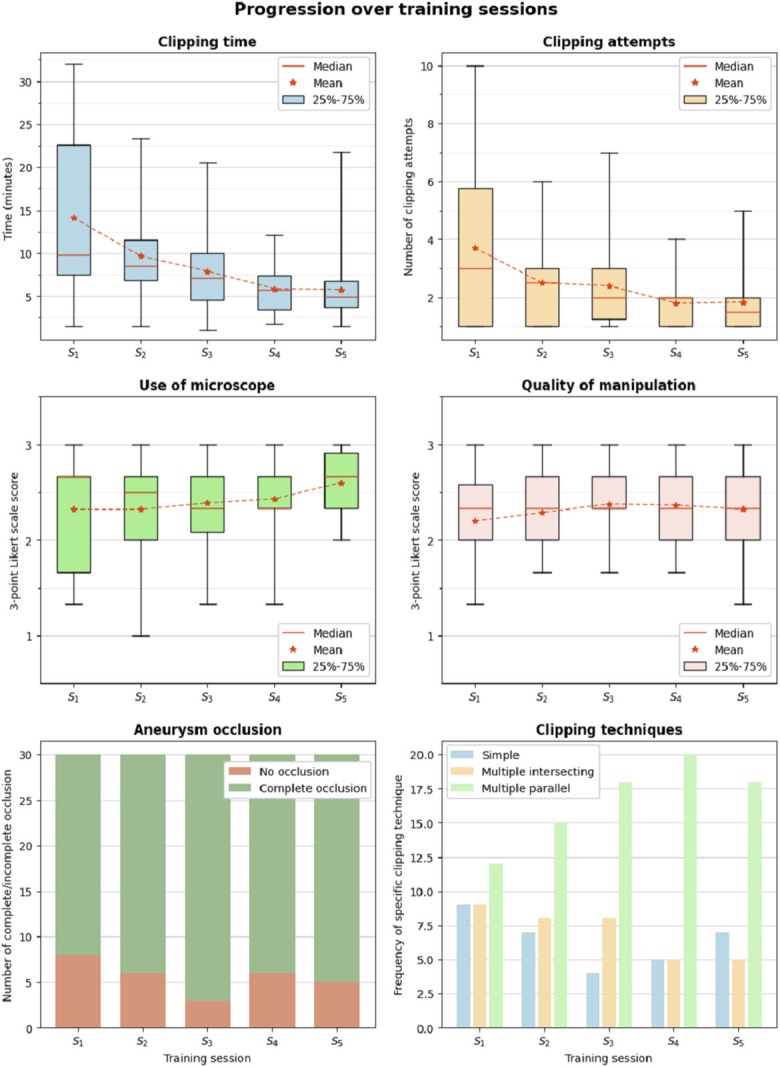
Overview of training performance parameters over simulation sessions.

Figure 5 shows the evolution of performance variables over training sessions. For each variable, all participants and PMs are included in each box plot or bar plot. The results of the linear regression fitting on the mean data are presented in Table 2. Clipping time and number of attempts decreased with increasing session count as the significant linear regression fitting proved an improvement in performance over time (*P* = .018, CI = [−204.65, −40.70] and *P* = .032, CI = [−0.81, −0.07], respectively). A substantial drop in clipping attempts occurred at session S_4_ immediately after the short mentoring session, where the suitability of different clipping techniques to approach different levels of complexity and anatomic characteristics was discussed. The use of microscope scoring also showed a significant correlation with clipping time (*P* = .027, CI = [0.02, 0.12]), with the largest improvement occurring at session S_5_, after the hands-on live teaching session. Quality of manipulation shows moderate improvement from S_1_ to S_3_, a period that included bibliographic review (S_1_ to S_2_), teaching IA clipping anatomic considerations, and surgical video learning (S_2_ to S_3_), illustrating didactical examples of a proficient technique in IA surgery. A slight decrease can be observed after the peak performance in session S_3_. Aneurysm occlusion increases from S_1_ to S_3_, and a decrease can be seen again toward S_4_ following the trend of using simple clipping techniques. Regarding clipping techniques, the multiple parallel technique was found to be the most popular clipping technique, and its use increased over training sessions.

**TABLE 2.** Linear Regression Fitting on the Mean of Performance Variables

Performance variable (Y)	Linear regression (Y = “slope” * “training session number” + “intercept”)					
	Slope	CI (slope)	*P* value (slope)	Intercept	CI (intercept)	*P* value (intercept)
Clipping time	−122.67	[−204.65, −40.70]	0.018	889.21	[617.32, 1161.09]	0.002
Clipping attempts	−0.44	[−0.81, 0.07]	0.032	3.78	[2.55, 5.00]	0.002
Use of microscope	0.07	[0.02, 0.12]	0.027	2.21	[2.04, 2.39]	0.000
Quality of manipulation	0.03	[−0.03, 0.09]	0.177	2.21	[2.02, 2.41]	0.000
Occlusion	0.60	[−1.20, 2.40]	0.367	22.60	[16.63, 28.57]	0.001

### Performance vs Model Difficulty

PMs were classified into two groups based on their morphology and difficulty to treat: PM_1_, PM_2_, and PM_5_ were grouped as the more challenging cases, whereas PM_3_ and PM_4_ were classified as the less challenging. The effects of the model grade of challenge on clipping time, number of clipping attempts, and quality of manipulation were expected to be relevant. Therefore, they were investigated through a series of 2-sample *t*-tests, as seen in Table 3. The results show that differences in clipping time (*P* = .0005, CI = [1.597, 5.611]) and the number of clipping attempts (*P* = .0152, CI = [0.157, 1.454]) between the more challenging group of models and the less challenging group are significant. The higher the grade of the challenge, the more the time and attempts are required. For its part, differences in the quality of manipulation scores showed a trend but were nonsignificant.

**TABLE 3.** Comparison of Clipping Time, Clipping Attempts, and Quality of Manipulation for Patient Model Difficulty

Model difficulty groups; Comparison statistics	Clipping time [min] Mean (SD)	Clipping attempts [attempts] Mean (SD)	Quality of manipulation [3-point Likert scale] Mean (SD)
More challenging (PM_1_, PM_2_, PM_5_)	10.13 (6.85)	3.09 (2.23)	2.26 (0.41)
Less challenging (PM_3_, PM_4_)	6.52 (4.61)	2.28 (1.45)	2.38 (0.28)
CI of the difference between groups	[1.597, 5.611]	[0.157, 1.454]	[−0.240, 0.002]
*P*-value of the two-sample *t*-test	0.0005	0.0152	0.0530

PM, patient model.

In addition, PM1, which is the most challenging PM, presented the highest rupture rate (50% of all rupture events (n = 8) corresponded to PM1) and the lowest rate of complete occlusion (33% of the cases for PM1 had no occlusion, compared with 13%–17% for the other PMs). Table 4 summarizes the clipping techniques used and the corresponding occlusion rates for each group. The less challenging group is treated more frequently with simple clipping techniques and less with multiple intersecting clipping techniques than the more challenging group. Lower occlusion rates can be observed for the more challenging cases.

**TABLE 4.** Comparison of Clipping Techniques and Aneurysm Occlusion for Patient Model Difficulty

Clipping technique	Less challenging cases (PM_3_, PM_4_)		More challenging cases (PM_1_, PM_2_, PM_5_)	
	Frequency of usage	% of aneurysm occlusion	Frequency of usage	% of aneurysm occlusion
Simple	19	84.21	13	69.2
Multiple intersecting	6	83.3	29	86.2
Multiple parallel	35	85.7	48	77.1

PM, patient model.

### Microscope Video Review

**FIGURE 6.**
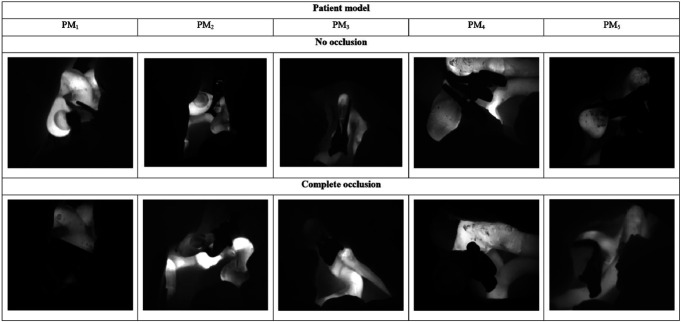
Examples of complete and incomplete occlusions per patient model captured during intraoperative indocyanine green-fluorescence angiography. PM, patient model.

An overview of occluded and non-occluded aneurysms, derived from the ICG microscope video recording, is presented per PM in Figure 6.

### Participant's Feedback

Quantitative (1-5 Likert scale) and qualitative participant feedback was collected during and after the study. Throughout the training sessions, the anatomic fidelity of the models for the radiological images was reported to be very high (mean: 4.33, SD: 0.54). In general, participants found the haptics of the vessels quite realistic although they agreed that the brain tissue could be a little softer (mean: 3.8, SD: 0.48). The blood flow and vessel pulsation were the highest rated features of the simulator (mean: 4.53, SD: 0.5). After 6 weeks of training, the participants found the simulator very useful for live hands-on teaching and mentoring (mean: 5.0, SD: 0) and for developing aneurysm clipping skills and increasing confidence (mean: 4.83, SD: 0.37). They also deemed it a promising technology for long-term training (mean: 4.5, SD: 0.76) and microsurgical skill evaluation purposes (mean: 4.5, SD: 0.5).

## DISCUSSION

On the one hand, according to the reduction in clipping time, number of clipping attempts, and the improvement in use of the microscope observed across the study, the residents show a learning curve through repetitive semi-independent training on the simulator, denoting familiarization with aneurysm clipping surgery and with the use of specific OT facilities. This matches the expectations and agrees with the results reported in similar studies with ex vivo models^29^ or VR^30^ simulators. Regarding research validity, the participant feedback collected is consistent with the findings of previous studies with the simulator.^21^

Repetitive training also allowed the residents to test different clipping techniques on the same model and get immediate feedback from the ICG-Fluorescence Angiography simulator option, enhancing their proficiency in a quantified manner with very minimal resources. On the other hand, the comparative analysis of performance between the two model difficulty groups suggests that the models can reproduce the complexity of real IA cases. Differences found between groups through two-sample *t*-testing in the number of clipping attempts and clipping time and observations made on the use of clipping techniques and corresponding occlusion rates reasonably align with expected difficulties in exposing and clipping the aneurysm because of the more complex anatomy of the more challenging cases.

Moreover, the relationship between certain distinguishable performance improvements with the complementary learning activities proposes the simulator as a tool of great added value to be integrated with conventional neurosurgery training curricula for the residents to practice their acquired theoretical knowledge and transform it into practical surgical abilities. However, this study does not intend to propose any specific set of learning methods to be included in a training program, and other complementary activities targeting broader or different skill areas may be found to produce greater effects on performance. Finally, among all the resources, the simulator performed well as a standalone device, being able to cover all elements of the simulation training (radiological examination of the patient case, positioning of the head model, inspection, and decision of clipping approaches preoperative and intraoperative actions, clipping, and intraoperative ICG-Fluorescence Angiography investigation) for several months (over 4 months). This indicates that training with the simulator can be run in a skill laboratory outside the OT if a surgical microscope and microsurgical instruments are available.^8,31-33^

## Limitations

Several limitations were encountered during the study. First, keeping a constant time interval between training sessions was challenging. This originated from an increased workload on the participants, who found it more difficult to fit S_4_ and S_5_ in their schedules (see **Supplemental Digital Content 4**, http://links.lww.com/ONS/B61: t_3 average_ = 9.2 days, t_4 average_ = 27.5 days) and often attended training sessions in their free time after long shifts or surgeries. This could be why the positive trend observed in the quality of manipulation up to the third session was interrupted, suggesting that frequent training included in the resident's working hours is required. Second, data were collected over a relatively short study duration (4 months). Third, although the performance indicators analyzed in this study have been extracted from existing performance scales specific to IA clipping,^22,26^ none of these scales have been used in their completely validated form because of incompatibilities with the current scope of the training models (no craniotomy and durotomy, no dissection, etc.) and to reduce the review time, which was known to be a time-consuming task.

In future research, an extended training period (during 1 or 2 years of the specialty residency) would allow for the analysis of long-term learning outcomes, where the effect of eventualities such as larger time gaps between sessions would be relativized. In turn, the need for more data warrants further progress in equipping simulators with an automated software-based systematic assessment tool. A machine computing model could result in uniform metrics, allowing the implementation of some of the existing objective scoring scales without concern for review time. However, from a technological point of view, developing such a model takes a lot of work to accomplish.

Finally, beyond residency training, after a retrospective feasibility study to determine whether the simulator can help in patient case preparation and management by anticipating challenges, difficulties, adverse events, and outcomes in clipping procedures, the next step would be to run a two-arm controlled prospective study using the simulator as a presurgical planning tool to determine the impact of training with the simulator on patient outcomes and on incidence of intraoperative adverse events and postoperative complications.

## Supplementary Material

**Figure:**
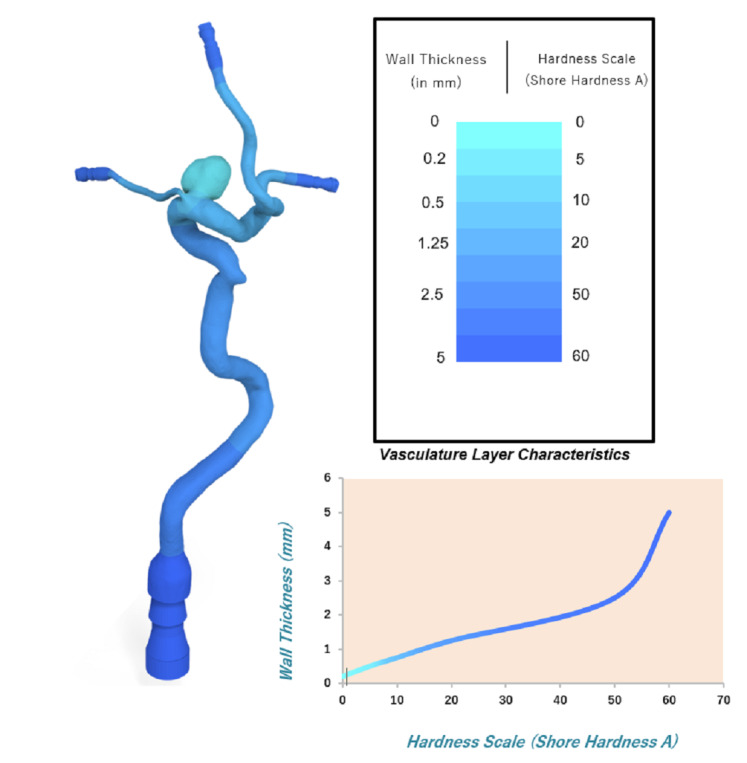


## Conclusion

This work shows that medical residents in neurosurgery can benefit from training with the proposed mixed-reality simulator to flatten the learning curve in IA clipping and the use of OT facilities such as the surgical microscope. Moreover, the simulator demonstrated the ability to replicate the complexity of real IA clipping cases. It acted robustly as a standalone device, allowing intensive training in a realistic environment throughout a mentoring process. Overall, the present findings strongly encourage further studies evaluating the integration of such a simulator into structured long-term training programs geared toward facilitating the way to board certification in neurosurgery and further development to spread these advancements to other neurosurgical disciplines and subspecialties.

## SUPPLEMENTAL DIGITAL CONTENT

**Supplemental Digital Content 1*,* Figure.** Technical specifications of the aneurysm model as received from the manufacturer (SurgeonsLab® AG, Switzerland). Wall thickness and elastic hardness of models across different regions of the artery model structure. **Supplemental Digital Content 2, Table.** Technical specifications of the set of surgical clips used. **Supplemental Digital Content 3, Methods.** Complementary Learning Activities. **Supplemental Digital Content 4, Table.** Intervals between each participant's consecutive training sessions during the study. **Supplemental Digital Content 5, Table.** Description of the scoring scale for microscope videos.

## COMMENTS

This paper described an exciting simulation system used for training neurosurgeons to complete intracranial aneurysm clipping. As many in the cerebrovascular field have bemoaned, aneurysm clipping is technically challenging and increasingly seldom performed in the era of endovascular neurosurgery, which leaves current trainees with less experience in such procedures at the time of graduation from residency or fellowship. The current rapid progress of endovascular innovations means that nearly all simple aneurysms currently have an endovascular treatment option, which selects ever more complicated and difficult cases for open treatment with fewer and fewer surgeons with the requisite experience to tackle them. The current state of the field mandates that neurosurgical training programs innovate their approach to cerebrovascular surgery, lest the traditional (and effective) techniques of microneurosurgery die out completely. The simulation system described in this paper is an exciting breakthrough in this regard. As the figures and text descriptions show, the authors were able to simulate real cases of MCA aneurysms based on catheter angiograms and provided an operating room-like environment including a neurosurgical microscope for residents to practice clip application. As expected, the post hoc analysis demonstrates that residents improved in speed and efficiency over time. This is an exciting innovation in neurosurgical training and should be applauded by academic neurosurgery and emulated wherever possible.

**Peter Theiss and Ali Alaraj**

*Chicago, Illinois, USA*
