# Supplementary material for: Training Performance Assessment for Intracranial Aneurysm Clipping Surgery Using a Patient-Specific Mixed-Reality Simulator: A Learning Curve Study
Source: Oper Neurosurg. 2024 Jan 22;26(6):727–36. doi: 10.1227/ons.0000000000001041 (PMC11086963; doi:10.1227/ons.0000000000001041)
Supplement: Supplementary file 1 [file ons-26-727-s001.docx]

**Supplemental Digital Content 2, Table:** Technical specifications of the set of surgical clips used.

| **Clip Type** | **Clip Size (Quantity)** | **Length [mm]** | **Geometry (Quantity)** | **Closing force [g]** | **Opening Width [mm]** |
| --- | --- | --- | --- | --- | --- |
| Permanent | Standard (131) | 4-15 | Straight (29)  Straight Fenestrated (3)  Elbow (42)  Elbow Fenestrated (3)  Bayonet (12)  Curved (41)  J-like (1) | 150-180 | 6.1-12.2 |
|  | Mini (59) | 3-7 | Straight (23)  Elbow (1)  Bayonet (1)  Curved (34) | 110 | 4.8-9.8 |
|  | Giant (1) | 25 | Straight (1) | 180 | 13.7 |
| Temporary | Standard (17) | 7-11 | Straight (11)  Bayonet (5)  Curved (1) | 90 | 8-10.2 |
|  | Mini (1) | 7 | Straight (1) | 70 | 7.1 |
