# Supplementary material for: Training Performance Assessment for Intracranial Aneurysm Clipping Surgery Using a Patient-Specific Mixed-Reality Simulator: A Learning Curve Study
Source: Oper Neurosurg. 2024 Jan 22;26(6):727–36. doi: 10.1227/ons.0000000000001041 (PMC11086963; doi:10.1227/ons.0000000000001041)
Supplement: Supplementary file 2 [file ons-26-727-s002.docx]

**Supplemental Digital Content 3, Methods:** Complementary Learning Activities.

**Abbreviations:** **S**, Session, **OT**, Operation Theater, **ICG-FA**, Indocyanine Green - Fluorescence Angiography.

Different complementary learning methods were implemented between each of the simulation training sessions as described below:

*Independent learning:* The residents completed two modalities of independent learning during the study:

1. Bibliographic learning (S_1_ to S_2_): Review of the chapters “Vascular Control”, “Temporary Clipping”, “Permanent Clipping” and “Inspection” from the book *Seven Aneurysms* ^1^, introducing the fundamentals of aneurysm clipping.
2. Surgical video learning (S_2_ to S_3_): Visualization of illustrative videos on clipping techniques for MCA aneurysms from the Aneurysm Clipping Video Atlas ^2^.

*Mentoring:* A senior cerebrovascular neurosurgeon was designated as a mentor to carry out personalized supervision of the learning progress of the study trainees. The supervision took place in two formats:

1. Short mentoring discussion (S_3_ to S_4_): Brief meeting (30–60 minutes), during which the mentor and the participants had access to the recorded microscopic and camera footage from their previous training sessions to discuss clipping approaches, clarify questions and identify opportunities for improvement.
2. Interactive live hands-on teaching session (S_4_ to S_5_): Mentoring session conducted with the simulator in the OT (seen in Fig. 2f). The mentor provided real-time feedback and advice during the treatment of two patient models (~60 minutes) and addressed a collection of topics relevant to neurosurgical training:
   1. Ergonomics and posture: Patient positioning strategies and recommendations to enable optimal access to the lesion and manipulation, specific to each aneurysm type and location.
   2. Use of instruments: Detailed explanations about the use of microsurgical instruments in different surgical activities.
   3. Radiological investigation: Review of basic concepts, such as localizing the lesion, and identifying surrounding structures, potential risks and challenges.
   4. Aneurysm clipping: General assistance throughout the procedure, including detailed feedback on specific moves or attempted strategies, and correction of errors or bad practices in real time.
   5. ICG evaluation: Insights on how to evaluate the outcome of the clipping in terms of aneurysm’s level of occlusion and neighboring vessels’ patency by means of intraoperative ICG-FA imaging.

**REFERENCES:**

1. Lawton MT. *Seven Aneurysms: Tenets and Techniques for Clipping*. 1st ed. Thieme; 2011.

2. Nussbaum ES. *Video Atlas of Intracranial Aneurysm Surgery*. 1st ed. Thieme; 2012.
