# Supplementary material for: Training Performance Assessment for Intracranial Aneurysm Clipping Surgery Using a Patient-Specific Mixed-Reality Simulator: A Learning Curve Study
Source: Oper Neurosurg. 2024 Jan 22;26(6):727–36. doi: 10.1227/ons.0000000000001041 (PMC11086963; doi:10.1227/ons.0000000000001041)
Supplement: Supplementary file 3 [file ons-26-727-s003.docx]

**Supplemental Digital Content 4, Table:** Intervals between each participant’s consecutive training sessions during the study

|  | **Resident ID and number of interval days (time interval – t_n_)** | | | | | |
| --- | --- | --- | --- | --- | --- | --- |
| **Session_Count_ (S)**  **t_n average_ in days** | **R_1_** | **R_2_** | **R_3_** | **R_4_** | **R_5_** | **R_6_** |
| **S_1_ to S_2_**  **(t_1 average_=6.7 days)** | 6 | 7 | 7 | 6 | 7 | 7 |
| **S_2_ to S_3_**  **(t_2 average_=7.2 days)** | 8 | 7 | 7 | 6 | 8 | 7 |
| **S_3_ to S_4_**  **(t_3 average_=9.2 days)** | 7 | 8 | 14 | 7 | 5 | 14 |
| **S_4_ to S_5_**  **(t_4 average_=27.5 days)** | 48 | 20 | 28 | 22 | 22 | 25 |
