# Supplementary material for: Training Performance Assessment for Intracranial Aneurysm Clipping Surgery Using a Patient-Specific Mixed-Reality Simulator: A Learning Curve Study
Source: Oper Neurosurg. 2024 Jan 22;26(6):727–36. doi: 10.1227/ons.0000000000001041 (PMC11086963; doi:10.1227/ons.0000000000001041)
Supplement: Supplementary file 4 [file ons-26-727-s004.docx]

**Supplemental Digital Content 5, Table:** Description of the scoring scale for microscope videos.

| **Knowledge unit** | **Score description** | | | |
| --- | --- | --- | --- | --- |
| **Use of microscope** | 1: Readjusts  positioning, focus and working distance frequently, frequently out of focus or using a magnification level that impedes proper field navigation | 2: Focused most of the time but readjusts at multiple instances, familiar with the use of microscope but not yet proficient | | 3: Optimizes zoom, focus, and optical settings at the beginning of task and adjusts only when needed |
| **Quality of manipulation** | 1: Poor technique, frequent damage of vessels, insufficient aneurysm exposure. | 2: Moderately good technique of dissection in proximity to vessels with acceptable or occasional accidental damage that does not affect the structural integrity of the vessel, adequate exposure of aneurysm and parent vessels. | | 3: Excellent technique of sharp and blind dissection, uninjured vessels, adequate and sufficient exposure of the aneurysm and parent vessels. |
| **Applied clipping technique** | Simple | Multiple intersecting | | Multiple parallel |
| **Aneurysm occlusion and remnant size** | No occlusion | | Complete occlusion | |
| **Aneurysm rupture** | No rupture | | Rupture | |
